# Holocentric Chromosomes Probably Do Not Prevent Centromere Drive in Cyperaceae

**DOI:** 10.3389/fpls.2021.642661

**Published:** 2021-02-19

**Authors:** Marie Krátká, Jakub Šmerda, Kateřina Lojdová, Petr Bureš, František Zedek

**Affiliations:** Department of Botany and Zoology, Faculty of Science, Masaryk University, Brno, Czechia

**Keywords:** asymmetric meiosis, centromere drive, CenH3, holocentric chromosomes, monocentric chromosomes, symmetric meiosis, meiotic drive

## Abstract

Centromere drive model describes an evolutionary process initiated by centromeric repeats expansion, which leads to the recruitment of excess kinetochore proteins and consequent preferential segregation of an expanded centromere to the egg during female asymmetric meiosis. In response to these selfish centromeres, the histone protein CenH3, which recruits kinetochore components, adaptively evolves to restore chromosomal parity and counter the detrimental effects of centromere drive. Holocentric chromosomes, whose kinetochores are assembled along entire chromosomes, have been hypothesized to prevent expanded centromeres from acquiring a selective advantage and initiating centromere drive. In such a case, CenH3 would be subjected to less frequent or no adaptive evolution. Using codon substitution models, we analyzed 36 CenH3 sequences from 35 species of the holocentric family Cyperaceae. We found 10 positively selected codons in the CenH3 gene [six codons in the N-terminus and four in the histone fold domain (HFD)] and six branches of its phylogeny along which the positive selection occurred. One of the positively selected codons was found in the centromere targeting domain (CATD) that directly interacts with DNA and its mutations may be important in centromere drive suppression. The frequency of these positive selection events was comparable to the frequency of positive selection in monocentric clades with asymmetric female meiosis. Taken together, these results suggest that preventing centromere drive is not the primary adaptive role of holocentric chromosomes, and their ability to suppress it likely depends on their kinetochore structure in meiosis.

## Introduction

During cell division, kinetochore assembly and microtubule attachment are typically limited to a small chromosomal region known as the centromere. However, this is not the case in organisms with holocentric chromosomes, in which CenH3 nucleosomes assemble (and microtubules thus bind) along the entire chromosome (Bureš et al., 2013). Holocentric chromosomes have originated independently at least 15 times in plants and animals (Melters et al., 2012; Drinnenberg et al., 2014; Escudero et al., 2016), but it is still unclear what evolutionary advantage allows the holocentric structure to arise and persist (Kolodin et al., 2018). One possible advantage of holocentric chromosomes might be the prevention of centromere drive (Malik and Henikoff, 2009; Talbert et al., 2009; Zedek and Bureš, 2016a).

Centromere drive model describes an evolutionary process during which “selfish” centromeres exploit asymmetric female meiosis to end up in the animal egg (or seed-plant megaspore, which is the only surviving meiotic product) and spread through the population (Henikoff et al., 2001). It begins with an expansion of the centromeric satellite array, which gains the ability to attract more CenH3 nucleosomes than its counterpart on the homologous chromosome. More CenH3 results in a “stronger” kinetochore, which preferentially captures microtubules of the egg pole during asymmetric female meiosis (Iwata-Otsubo et al., 2017; Akera et al., 2019). The unconstrained drive of the selfish centromere can undermine organismal fitness by spreading harmful hitchhiking mutations or causing nondisjunction during male meiosis (Malik and Henikoff, 2009). These harmful effects can be countered by the adaptive evolution of kinetochore proteins such as CenH3 (Malik and Henikoff, 2009; Finseth et al., 2020). Mutations that produce a CenH3 variant binding to the driving and the regular centromere with the same affinity, thereby balancing the kinetochore on both centromere variants, could be positively selected (Malik and Henikoff, 2009; Finseth et al., 2020). Over the last couple of years, the evidence supporting the centromere drive model has slowly been gathering (reviewed for instance in Lampson and Black, 2017 and Kursel and Malik, 2018). Briefly summarized, (i) it has been shown that centromeric satellites can affect the positioning of CenH3 (Zhang et al., 2013; Akera et al., 2017) and that centromeres with more satellite repeats recruit more CenH3 and increase their transmission to the egg relative to homologous centromeres with fewer repeats (Iwata-Otsubo et al., 2017); (ii) the meiotic spindle asymmetry (the key assumption) based on differential tyrosination of microtubules (emanating from polar body and egg pole) has been directly linked with size-dependent centromere competition (Akera et al., 2017) and recently even characterized on the molecular level (Akera et al., 2019); (iii) the negative effects of centromere drive on fitness have been documented in monkeyflowers population (Fishman and Kelly, 2015; Finseth et al., 2020), which was shown (iv) to be counterbalanced by selective sweep in CenH3, thus proving evolutionary arms race between selfish centromeres and the key kinetochore protein (Finseth et al., 2020).

Centromere drive can occur in lineages where centromeric sequence expansion causes changes in the kinetochore “strength” and where asymmetric meiosis allows the “stronger” centromere to be preferentially segregated to the gamete. However, in lineages, where both male and female meiosis are symmetric (gametes originate from all four meiotic products), such as fungi or cryptogamous plants, the “stronger” centromere does not have any advantage and centromere drive should not occur (Talbert et al., 2009; Zedek and Bureš, 2016b). Consequently, CenH3 in lineages with exclusively symmetric meiosis is subject to positive selection with a lower frequency than in lineages with asymmetric meiosis (Zedek and Bureš, 2016b).

A similar situation may arise in holocentric organisms. The chromosome sites associated with CenH3 nucleosome recruitment in holocentrics rarely consist of specific satellite repeats (Plohl et al., 2014; Marques et al., 2015), so possibly, the CenH3 location is not closely tied to the underlying sequence (or such sequences, other than satellite repeats, have not yet been recognized). With CenH3 recruitment independent of the sequence, it is unlikely that expansion of the satellite array would initiate centromere drive (Bureš and Zedek, 2014). In accordance with this hypothesis, no signs of positive selection acting on CenH3 were found in the holocentric plant genus *Luzula* (Juncaceae; Zedek and Bureš, 2016a). This suggests that holocentric chromosomes prevent centromere drive in *Luzula* (Zedek and Bureš, 2016a). However, in the sister holocentric family Cyperaceae, repeat-based holocentromeres have been reported in the genus *Rhynchospora*, in which the CenH3 position and the kinetochore formation are colocalized with a centromeric satellite repeat called Tyba in mitosis and meiosis (Marques et al., 2015, 2016). While in mitosis kinetochore is formed along the length of chromosomes in *Rhynchospora* (Marques et al., 2015), in meiosis kinetochore is restructured and forms several separate clusters (Marques et al., 2016). Moreover, not only female, but also male meiosis is asymmetric in Cyperaceae and thus only a single viable gamete is retained while the other three haploid nuclei degenerate during pollen meiosis (Furness and Rudall, 2011; Rocha et al., 2016). The clustered meiotic kinetochore formation in *Rhynchospora* may possibly allow centromere drive to occur because a space is left for the kinetochore enlargement in response to expanding underlying repeats. The asymmetric meiosis in both sexes of Cyperaceae species may, in turn, provide more opportunities for centromere drive to occur in this family.

The centromere drive model predicts that in clades where centromere drive occurred, frequency of positive selection acting on CenH3 should be higher than in clades without opportunities for centromere drive (Zedek and Bureš, 2016b). Therefore, in this study, we aim to quantify the frequency of positive selection acting on CenH3 in representatives of the Cyperaceae family and compare it to frequencies of positive selection in various holocentric and monocentric taxa. This could show the potential effect of centromere drive on the holocentric Cyperaceae family and further elucidate whether the holocentric chromosome structure might have evolved as a defense against centromere drive.

## Materials and Methods

### Obtaining the CenH3 Sequences

Species of the Cyperaceae family were collected from wild populations (in Brno and Žďárské vrchy, Czechia), from the collection of the Department of Botany and Zoology of Masaryk University, and the private collection of Pavel Veselý. The list of coordinates and sources of the plant material is supplied in Supplementary Table S1. The bases of young leaves were ground to a fine powder in liquid nitrogen, and total genomic RNA was isolated using an RNeasy Plant Mini Kit (QIAGEN) according to the manufacturer’s instructions. RNA was then transcribed to cDNA using a QuantiTect Reverse Transcription Kit (QIAGEN) with universal primers according to the manufacturer’s instructions. Sequences of the CenH3 cDNA were then amplified using PCR with specific primers. Thermocycler parameters and sequences of the primers can be found in Supplementary Tables S2 and S3. Products of the reactions were analyzed electrophoretically on a 1% agarose gel. Products were then sequenced by Macrogen, Inc., Netherlands. Sites, where multiple nucleotides were equally plausible based on the sequencing chromatogram, were symbolized with the use of degenerate nucleotide symbols. Additional sequences were obtained from the GenBank database. GenBank accessions of all the analyzed sequences can be found in Supplementary Table S1.

### Sequence Analysis

The inference of selection regimes acting on CenH3 in Cyperaceae was based on application of codon substitution models on sequences aligned at codon level. Codon substitution models infer the selective pressures acting on a protein/gene from the non-synonymous/synonymous substitution rate ratio (dN/dS = *ω*). Non-synonymous substitutions change the amino acid coded by the respective codon, while synonymous substitutions do not. In case of no selective pressure (neutral evolution), non-synonymous and synonymous substitutions occur at the same rate (*ω* = 1). Purifying selection is indicated by *ω* < 1, and positive selection favoring substitutions that change the amino acids in a protein are indicated by *ω* > 1.

To allow the comparison of positive selection frequencies in Cyperaceae with 19 eukaryotic clades, we analyzed earlier (Zedek and Bureš, 2016a,b), we applied the same procedures. Codon alignment and phylogenetic tree topology were inferred in BAli-Phy 3.4.1 (Redelings and Suchard, 2005) using the GY94 substitution and M0 indel models and running five independent chains with 100,000 iterations and 10% burn-in. For every aligned codon, BAli-Phy also calculates the reliability score (from 0 to 100%), which is the probability that a given codon should indeed be placed at a given site in the alignment. Codons aligned with a reliability score below 80% were treated as missing data and masked as “???” (one “?” for each nucleotide in the codon). Because aBS-REL and MEME – the algorithms we used for positive selection analyses (see below) – treat all gaps as missing data, we coded all the external and internal gaps in the alignment as “?”s as well. The non-masked alignment in the fasta format is available in Supplementary Table S4.

The masked alignment (Supplementary Table S5) and 50% consensus tree (Supplementary Table S5) were then analyzed using aBS-REL (Smith et al., 2015) and MEME (Murrell et al., 2012) codon substitution models to identify positively selected phylogenetic branches and codon positions, respectively. We used the implementation of these models on the Datamonkey 2.0 webserver (Weaver et al., 2018). Although missing data cannot generate false positives in aBS-REL and MEME analyses, they may reduce the power to detect positive selection. However, this is not a problem in our case, because our main goal is to determine the frequency of positively selected branches and codons in CenH3 of Cyperaceae and then compare these relative measures with 19 other eukaryotic clades that were analyzed previously in the very same way (Zedek and Bureš, 2016b). Tree lengths in the number of nucleotide substitutions per codon site were obtained using PAML 4.7 (Yang, 2007) with the M0 model of codon substitutions. The proportion of positively selected branches (respective codons) in the family Cyperaceae was then compared with other holocentric and monocentric taxa. Sequences were obtained from Zedek and Bureš (2016a,b). Because the algorithm previously used for the detection of positively selected branches was BS-REL and not aBS-REL (Zedek and Bureš, 2016a,b), we re-analyzed all the previous clades with aBS-REL to allow proper comparison.

## Results

**Figure 1:**
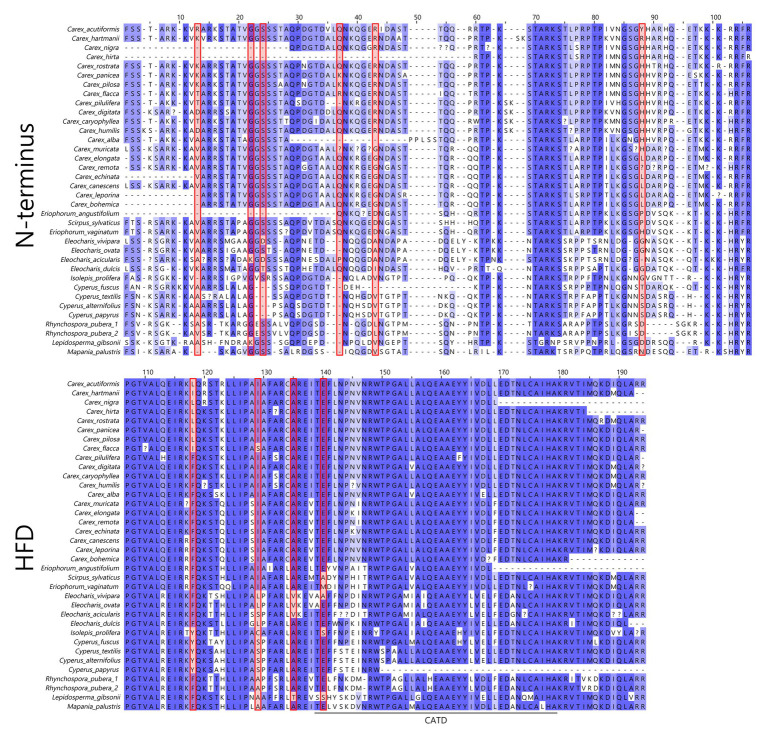
Non-masked amino-acid alignment of Cyperaceae CenH3. Positions of positively selected codons in CenH3 are marked by red rectangles. The numbers of amino acids correspond to the numbers of codons from the MEME output (Supplementary Table S6). HFD, histone fold domain; CATD, centromere targeting domain.

**Figure 2:**
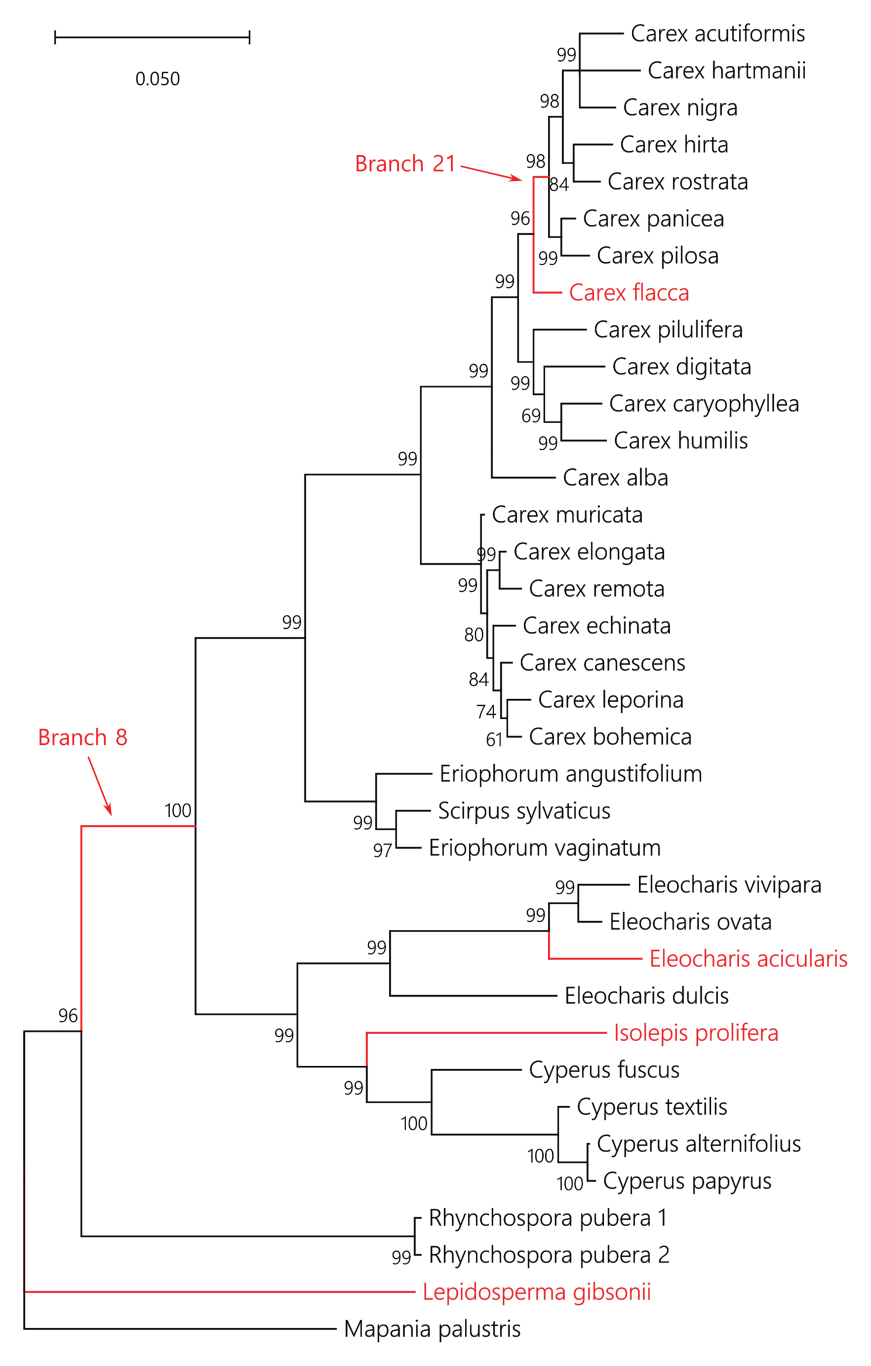
Phylogenetic tree of analyzed CenH3 sequences with six branches under positive selection depicted in red. The numbers above or below branches are statistical supports in Bayesian posterior probabilities. The aBS-REL output containing the exact *p* values of positive selection tests for each branch is available in Supplementary Table S7.

**Figure 3:**
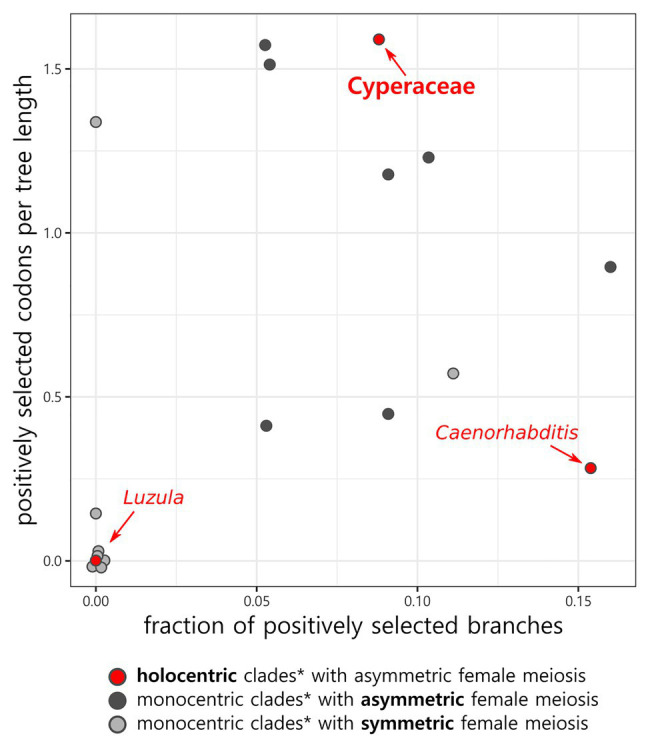
Fraction of positively selected branches and number of positively selected codons relative to the tree length in CenH3 in Cyperaceae and other groups. Jitter has been applied to the data points to differentiate the overlapping points at the origin. ^*^More detailed information on genera, families, and is listed in Table 1.

In total, 36 CenH3 sequences from 35 species of Cyperaceae were analyzed. MEME identified 10 positively selected codons (*p* ≤ 0.05; Supplementary Table S6). Six positively selected codons (codons no. 13, 22, 24, 37, 43, and 88) were in the N-terminal tail of the protein (Figure 1). Four positively selected codons (codons no. 117, 129, 134, and 140) were in the histone fold domain (HFD), including one codon (codon no. 140) in the loop-1 region of the centromere targeting domain (CATD; Figure 1), which directly interacts with the DNA (Dalal et al., 2007). No branches were found to be positively selected at the *p* ≤ 0.05 significance level after Holm-Bonferroni multiple testing corrections (Supplementary Table S7). However, when aBS-REL is used for explanatory analyses (i.e., for testing all branches of a given phylogeny), correction of multiple testing is not appropriate, because it substantially reduces power with the growing number of branches, while the amount of statistical signal does not increase (Spielman et al., 2019). Without correction, six positively selected branches were found (Figure 2), namely four tip branches of *Lepidosperma gibsonii*, *Isolepis prolifera*, *Eleocharis acicularis*, and *Carex flacca*, and two internal branches (branches no. 8 and 21). The analysis of positive selection frequency showed the number of positively selected codons per tree length was 1.766 and the frequency of positively selected branches (before multiple testing corrections) was 8.8%. Figure 3 shows the positive selection frequency in Cyperaceae’s CenH3 in the context of CenH3 from other 19 eukaryotic clades comprising clades with monocentric and holocentric chromosomes as well as clades with (asymmetric meiosis) or without (symmetric meiosis) opportunities for centromere drive. The numbers upon which Figure 3 is based are shown in Table 1. This comparison showed that the frequency of positive selection acting on the CenH3 gene in Cyperaceae is similar to that in lineages with monocentric chromosomes and asymmetric female meiosis, where centromere drive takes place (Zedek and Bureš, 2016b).

**Table 1:** Fraction of positively selected branches and codons in Cyperaceae and other clades.

Clade	Type^1^	Number of sequences	Tree length	Branches^2^	Branches corr^3^	Codons^4^
*Aspergillus*	M/S	18	8.725	0	0	0
*Colletotrichum*	M/S	7	1.096	0	0	0
Ferns	M/S	8	7.587	0	0	0
Lycopodiophyta	M/S	5	6.045	0	0	0
Bryophyta	M/S	10	2.242	0	0	1.338
*Penicillium*	M/S	11	5.814	0.105	0	0
*Plasmodium*	M/S	7	3.247	0	0	0
*Saccharomyces*	M/S	9	6.921	0	0	0.144
*Trichoderma*	M/S	6	1.75	0.111	0	0.571
Asteraceae	M/A	7	1.698	0.091	0	1.178
Brassicaceae	M/A	20	3.179	0.053	0	1.573
*Drosophila*	M/A	16	4.065	0.103	0.069	1.230
Fabaceae	M/A	18	4.464	0.091	0.030	0.448
Bony Fish	M/A	11	4.856	0.053	0	0.412
Poaceae	M/A	20	3.965	0.054	0	1.513
Primates	M/A	14	1.116	0.16	0	0.896
*Tetrahymena*	M/A	13	8.465	0.043	0.043	0.118
*Caenorhabditis*	H/A	8	7.074	0.154	0	0.283
*Luzula*	H/A	18	1.278	0	0	0
Cyperaceae	H/A	36	5.662	0.088	0	1.590

1 Holocentric (H) or monocentric (M) chromosomes, asymmetric (A) or symmetric (S) female meiosis.

2 Fraction of positively selected branches in the phylogenetic tree at the *p* < 0.05 level without multiple testing corrections.

3 Fraction of positively selected branches in the phylogenetic tree at the *p* < 0.05 level with multiple testing corrections.

4 Number of positively selected codons divided by the tree length.

## Discussion

Our phylogenetic tree of Cyperaceae CenH3 is congruent with the species phylogenies of Cyperaceae based on multiple nuclear and chloroplast markers (Márquez-Corro et al., 2019; Semmouri et al., 2019), suggesting that our dataset does not contain paralogous sequences resulting from ancient duplications. It is possible that our primers simply did not recognize potential paralogs. However, the CenH3 sequences that we obtained from GenBank (Supplementary Table S1) come from transcriptome sequencing, so the absence of ancient paralogs due to our primers is unlikely. We cannot distinguish, however, whether the two CenH3 variants from *Rhynchospora pubera* are recent paralogs or mere polymorphism of a single locus. But as none of the two variants was positively selected, we may conclude that the positive selection acting on CenH3 in Cyperaceae did not arise from paralogs adopted for new functions.

We detected multiple episodes of positive selection acting on CenH3 that have occurred across the gene (Figure 1) and its phylogeny (Figure 2) indicating that CenH3 in Cyperaceae has been subjected to recurrent adaptive evolution. The frequency of positive selection events is comparable to eukaryotic lineages in which the centromere drive can act (Figure 3; Table 1). We have also identified four positively selected codons in the HFD including one codon on the loop-1 region of the CATD (Figure 1), which is necessary for CenH3 targeting to the centromere (Dalal et al., 2007). These results suggest that CenH3 in Cyperaceae may be in arms-race with selfish repeats in the process of centromere drive. Such findings are not consistent with the absence of positive selection acting on CenH3 in the holocentric genus *Luzula* (Zedek and Bureš, 2016a) and contradict the hypothesis that holocentric chromosomes prevent centromere drive.

The stark difference between selection patterns of CenH3 in Cyperaceae compared with *Luzula* could stem from the diversity of meiotic chromosome behavior across holocentric lineages (Marques and Pedrosa-Harand, 2016), which arises as a reaction to the inherent problems with meiotic chromosome segregation in holocentrics (Melters et al., 2012; Marques and Pedrosa-Harand, 2016). One possible solution to these problems is confining kinetic activity to chromosomal ends in meiosis, as observed in nematodes or true bugs (Marques and Pedrosa-Harand, 2016). Such a change of the mitotic kinetochore spanning the length of chromosomes to the localized meiotic kinetochore, which resembles monocentric kinetochore, could possibly allow centromere drive to occur by providing space for kinetochore enlargement. Accordingly, CenH3 in *Caenorhabditis* shows signs of positive selection comparable to that of monocentric lineages with asymmetric meiosis (Figure 3; Zedek and Bureš, 2012, 2016b). Another solution is to invert the order of meiotic events in such a way that sister chromatids segregate in the first meiotic division, while homologs segregate in the second, as documented in *Luzula* from Juncaceae (Heckmann et al., 2014) or *Rhynchospora* from Cyperaceae (Cabral et al., 2014; Marques et al., 2016). However, although both *Luzula* and *Rhynchospora* employ inverted meiosis, their meiotic kinetochores differ (Marques and Pedrosa-Harand, 2016).

While in *Luzula*, the meiotic kinetochore is formed along the chromosome in a holocentric fashion, the meiotic kinetochore of *Rhynchospora* forms polycentric separated clusters (Marques et al., 2016). Moreover, CenH3 of *R. pubera* colocalizes with specific centromeric repeats named Tyba both in mitosis and meiosis (Marques et al., 2015, 2016), while in *Luzula* kinetochore formation is independent of satellite repeats (Heckmann et al., 2013; Jankowska et al., 2015). The clustered meiotic kinetochore of *Rhynchospora* still leaves some space for its enlargement and could therefore change its size in response to expanding underlying satellite repeats and allow centromere drive to occur. In such a case, centromere drive would merge with the holokinetic drive (Bureš and Zedek, 2014), because each expansion of these repeats would also lead to an enlargement of the respective chromosome. Although the repeat-based centromeres and the clustered meiotic kinetochore could explain the frequent adaptive evolution of CenH3 in Cyperaceae (Figure 3), it is yet to be discovered whether it is a common feature of the entire family or just a specific case of *Rhynchospora*. The clustered CenH3 distribution was recently observed in mitosis of the holocentric *Cuscuta europea* (Convolvulaceae), but microtubule attachment was independent on CenH3 in this species and occurred along the entire chromosomes (Oliveira et al., 2020). CenH3-independent kinetochore formation occurs also in holocentric insects in which this histone protein is entirely missing (Drinnenberg et al., 2014) and in *Bombyx mori* (Lepidoptera), its function is replaced by CENP-T (Cortes-Silva et al., 2020). CenH3 also appears absent in *Aldrovanda vesiculosa* and *Drosera spatulata* from the holocentric family Droseraceae (see Figure S5 in Palfalvi et al., 2020). The independent losses of CenH3 or its function accompanying transitions to holocentricity suggest that when holocentric chromosomes evolve, CenH3 may become dispensable (Zedek and Bureš, 2016a) or even a burden. Unlike monocentrics, holocentric organisms seem to have very homogenous chromatin with no clear distinction of eu‐ and heterochromatin (Mandrioli and Manicardi, 2012, 2020; Heckmann et al., 2013) and at least in *Caenorhabditis* and *Bombyx mori* kinetochore formation avoids transcriptionally active chromatin (Gassmann et al., 2012; Cortes-Silva et al., 2020). It may be that the chromatin restructuralization connected with transition to holocentricity eventually leads to the loss of CenH3. And the present-day holocentric clades may be in different phases of this process; holocentric insects and some Droseraceae have already lost CenH3, *Cuscuta* has CenH3 but its kinetochore formation and microtubules attachment is CenH3-independent, *Luzula*’s kinetochore still appears CenH3-dependent, but selective pressures on its CenH3 may already be relaxed (Zedek and Bureš, 2016a) and in Cyperaceae, kinetochore formation appears fully CenH3-dependent and CenH3 in this family may thus contribute to defense against centromere drive.

Another factor contributing to centromere drive in Cyperaceae, which is not present in *Luzula*, might be the asymmetry of both female and male meiosis. In this case, three out of four meiotic products during pollen development also undergo abortion (a process termed monomicrospory; Furness and Rudall, 2011; Rocha et al., 2016), although the exact mechanism and the timing of events differ from asymmetric female meiosis (San Martin et al., 2013; Rocha et al., 2016). The meiotic asymmetry in both sexes of Cyperaceae (Furness and Rudall, 2011; Rocha et al., 2016) increases the potential for selfish centromeres to gain an advantage in both meioses, as probably occurs in the ciliate genus *Tetrahymena* (Elde et al., 2011). To further discern the effect of monomicrospory on the adaptive evolution of CenH3, it might be preferable to study Ericaceae (a dicot family with monocentric chromosomes), where species with symmetric and asymmetric male meiosis exist side-by-side (Furness and Rudall, 2011).

An alternative explanation for the frequent positive selection acting on CenH3, we observed in Cyperaceae (Figure 3; Table 1) may be that some Cyperaceae species are actually monocentric. The example of *Cuscuta*, where holocentric and monocentric chromosomes are found within a single genus (Pazy and Plitmann, 1994; Neumann et al., 2020; Oliveira et al., 2020) shows that this possibility cannot be ruled out. Cyperaceae comprises 5,695 species (The Angiosperm Phylogeny Group, 2016), but chromosomes were counted in 1,140 species (~20%; Rice et al., 2015) and more closely inspected regarding chromosome structure in roughly 100 species (~1.8%; 78 species listed by Melters et al., 2012 plus some other inspected later). Contrary to the previous report (Zedek et al., 2016), monocentric chromosomes were recently proven by CenH3 and microtubule immunolabeling in mitosis of *Prionium serratum* from a small related cyperid family Thurniaceae (Baez et al., 2020). Monocentric chromosomes were also suggested in four species of the genus *Juncus* from the sister family Juncaceae (Guerra et al., 2019), although the authors base their conclusions on DAPI and CMA staining and histone phosphorylation patterns (Guerra et al., 2019), none of which are reliable markers for holo‐ or monocentricity (Kolodin et al., 2018; Neumann et al., 2020). On the other hand, although we cannot be entirely sure that all Cyperaceae are holocentric, so far, there is no evidence to the contrary. Moreover, we have many reasons to consider them holocentric. Holocentric chromosomes have been proven by inheritance of induced fragments, *via* CenH3 labeling or ultrastructural microscopy at least in *Eleocharis* (Hakansson, 1954), *Rhynchospora* (Marques et al., 2015), and *Cyperus* (Braselton, 1971). Most Cyperaceae genera, especially *Carex* and *Cyperus*, show extreme variation in chromosome number (species of *Carex* showing all the chromosome numbers from *n* = 6 to *n* = 56), most likely caused by their tolerance to chromosome fragmentation (Bureš et al., 2013). Cyperaceae (e.g., *Carex*, *Bulbostylis*, and *Kobresia*) are also extremely resistant to ionizing radiation (causing chromosome breaks) and highly competitive in chromosome-breaking conditions (reviewed in Zedek and Bureš, 2018). Although basal Cyperaceae taxa as Lepidosperma or Mapania have not been studied in detail and may thus be monocentric, most of the positively selected branches that we detected (Figure 2) are from the taxa, in which the existing evidence point to holocentric chromosomes.

Frequent adaptive evolution of CenH3 in Cyperaceae suggests that the evolution of centromeric repeats and thus centromere drive may occur in this holocentric family. It is therefore unlikely that holocentric chromosomes originated in the common ancestor of Cyperaceae and Juncaceae as an adaptation to deal with centromere drive. Since all organisms with holocentric chromosomes have the same chromosomal structure during mitosis but differ during meiosis (Marques and Pedrosa-Harand, 2016), it seems that the primal advantage of holocentric chromosomes is the tolerance to fragmentation in mitosis (Zedek and Bureš, 2018, 2019). Once holocentric chromosomes arise, they impose segregation problems in meiosis (Melters et al., 2012). Some ways of dealing with these problems preserve kinetochore assembly along the entire chromosome (genus *Luzula*), and it appears that this structure prevents centromere drive (Zedek and Bureš, 2016a). However, this is not universal for all holocentric organisms (Marques and Pedrosa-Harand, 2016), and it would be desirable to study the relationship between meiotic chromosomal structure and centromere drive in additional holocentric genera such as *Drosera*, *Cuscuta*, or *Chionographis* to fully reconcile the role of holocentric chromosomes in centromere drive suppression.

## Data Availability Statement

Data supporting the findings of this work are available within the article and its Supplementary Material. The CenH3 sequences generated and analyzed during the current study are available in Genbank at: https://www.ncbi.nlm.nih.gov/genbank/, accessions MN540151–MN540179.

## Author Contributions

MK performed lab work and bioinformatic analyses, contributed to sample collection, and co-drafted the manuscript. JŠ and KL participated in the lab work and sample collection. PB contributed to the design of the study, collected most of the samples, and helped with writing the manuscript. FZ designed the study, contributed to lab work and bioinformatic analyses, and co-drafted the manuscript. All authors contributed to the article and approved the submitted version.

### Conflict of Interest

The authors declare that the research was conducted in the absence of any commercial or financial relationships that could be construed as a potential conflict of interest.
